# Sepsis-induced cardiomyopathy: mechanisms, epidemiology, diagnosis, and treatments

**DOI:** 10.3389/fimmu.2026.1785463

**Published:** 2026-03-11

**Authors:** Fei Cao, Tao Yan, Chaoyue Liang, Ao Liu, Junyan Wang

**Affiliations:** 1 Department of Critical Care Medicine, Baotou Central Hospital, Baotou, Inner Mongolia, China; 2 Department of Critical Care Medicine, The First Affiliated Hospital of Baotou Medical College, Inner Mongolia University of Science and Technology, Baotou, Inner Mongolia, China

**Keywords:** sepsis-associated cardiomyopathy, inflammatory response, epidemiology, diagnosis, treatment

## Abstract

Sepsis-induced cardiomyopathy (SICM) is a life-threatening complication of sepsis characterized by reversible myocardial dysfunction. Its pathophysiology involves a complex interplay of inflammatory pathways, oxidative stress, mitochondrial dysfunction, and genetic factors, leading to impaired cardiac contractility, ventricular dilatation, and increased mortality. Epidemiologically, SICM exhibits demographic variations, with younger age, male sex, and pre-existing heart failure identified as key risk factors. Diagnostic approaches rely on a combination of biomarkers (e.g., troponins, NT-proBNP), advanced imaging techniques (e.g., speckle-tracking echocardiography), and clinical criteria, though standardized definitions remain elusive. Treatment strategies include pharmacological interventions (e.g., levosimendan, melatonin), mechanical circulatory support (e.g., VA-ECMO, Impella), and emerging therapies targeting ferroptosis or mitochondrial function. Historical perspectives highlight the evolution from recognizing SICM as a rare complication to its current status as a major contributor to sepsis mortality, while future directions emphasize personalized medicine, multi-omics integration, and targeted therapeutics. This review synthesizes current knowledge of SICM, identifies critical gaps, and proposes actionable research priorities to improve patient outcomes.

## Introduction

Sepsis, defined as life-threatening organ dysfunction caused by a dysregulated host response to infection (Sepsis-3), is a global health challenge associated with high mortality (1). Sepsis-induced cardiomyopathy (SICM) is a frequent and serious cardiac complication, characterized by acute, often reversible, biventricular systolic and diastolic dysfunction in the absence of other cardiac causes (2, 3). Epidemiological studies suggest that SICM occurs in 13.8% to 50.3% of sepsis patients, with significantly higher mortality rates (3). Its pathophysiology involves a complex interplay of systemic inflammation, mitochondrial dysfunction, calcium dysregulation, and microcirculatory disturbances (4–6).

Diagnosis remains challenging, relying on echocardiographic findings such as reduced left ventricular ejection fraction in the context of sepsis, often requiring exclusion of fluid overload and other cardiopathies (7, 8). Current treatment is primarily supportive, focusing on sepsis source control, judicious fluid management, and first-line vasoactive agents like norepinephrine, with inotropes considered in specific scenarios (9–11). The lack of specific biomarkers and unified diagnostic criteria complicates both clinical management and research. This review aims to synthesize current knowledge on SICM, exploring its mechanisms, epidemiology, diagnostic approaches, therapeutic strategies, and future directions to improve patient outcomes.

## Sepsis-induced cardiomyopathy: pathophysiological mechanisms

### Inflammatory pathways in sepsis-induced cardiomyopathy

The inflammatory response in SICM is driven by the dysregulated activation of innate immune pathways, particularly the NF-κB signaling cascade, which mediates the release of pro-inflammatory cytokines such as TNF-α, IL-1β, and IL-6 (12, 13). In a murine model of lipopolysaccharide (LPS)-induced sepsis, simvastatin pretreatment reduced myocardial NF-κB p65 expression by 35%, respectively, leading to a 50% decrease in TNF-α and IL-6 levels and improved left ventricular ejection fraction (LVEF) from 34% to 56% (12). Similarly, apelin-13, a peptide hormone, attenuated LPS-induced cardiac dysfunction in rats by inhibiting NF-κB nuclear translocation, resulting in a 60% reduction in myocardial TNF-α levels and a 2.5-fold increase in survival rate (14). The cholinergic anti-inflammatory pathway (CHAIP) also plays a critical role: GTS-21, an α7-nicotinic acetylcholine receptor (α7nAChR) agonist, reduced sepsis-induced myocardial injury in mice by 70% via suppression of NF-κB and STAT3 activation (15). Additionally, the NLRP3 inflammasome contributes to SICM pathogenesis: inhibition of NLRP3 in CLP-induced septic mice reduced cardiac caspase-1 activity by 55% and IL-1β levels by 60%, leading to a 30% improvement in LVEF (16). These pathways are interconnected: sulfur dioxide (SO_2_) donor treatment in septic rats inhibited TLR4/NLRP3 signaling, reducing myocardial Caspase-1 expression by 45% and improving cardiac output by 25% (17). Collectively, these data demonstrate that targeting inflammatory signaling cascades—including NF-κB, CHAIP, and NLRP3—can mitigate SICM severity.

Complement activation and extracellular histones further exacerbate inflammatory injury in SICM. In polymicrobial sepsis, complement component C5a induces neutrophil extracellular trap (NET) formation, releasing histones that reduce myocardial levels of SERCA2, NCX, and Na^+^/K^+^-ATPase by 30–40% (18). A retrospective study demonstrated that elevated plasma histone H4 levels (0.26 mg/L vs. 0.22 mg/L in non-SICM patients) were independently associated with SICM patients development (OR = 6.502, 95% CI: 1.203–78.231, p = 0.044) and increased ICU mortality (40.0% vs. 19.7% in non-SICM patients) (19). Moreover, the TLR4-MyD88 pathway mediates inflammatory cell infiltration: FAM46C overexpression in LPS-treated AC16 cells and c57 mice reduced ICAM-1 and VCAM-1 levels by 50% and 45%, respectively, via inhibition of p38 and ERK/MAPK signaling, leading to a 35% decrease in apoptotic cell death (20). These findings highlight the multifaceted nature of inflammatory injury in SICM and the need for targeted anti-inflammatory strategies.

### Role of oxidative stress in sepsis-induced cardiomyopathy

Oxidative stress is a central mediator of SICM, driven by increased reactive oxygen species (ROS) production and impaired antioxidant defense mechanisms. NADPH oxidase 2 (NOX2) activation is a key source of mitochondrial superoxide in septic cardiomyocytes: LPS treatment increased NOX2 expression by 2.3-fold in isolated adult mouse cardiomyocytes, leading to a 40% reduction in mitochondrial membrane potential and a 25% decrease in calcium transient amplitude (21). Genetic ablation of NOX2 preserved LVEF (60% vs. 45% in wild-type mice) and reduced myocardial ROS levels by 55% in CLP-induced septic mice (21). Mitochondrial dysfunction exacerbates oxidative stress: Drp1/Fis1-mediated mitochondrial fission in LPS-treated H9C2 cells increased mitochondrial ROS by 60%, while inhibition of Drp1/Fis1 interaction with P110 normalized membrane potential and improved cardiac function in mice (22). Additionally, impaired SIRT3 activity due to NAD^+^ depletion contributes to oxidative injury: LPS-induced NAD^+^ depletion in murine hearts reduced SIRT3 deacetylase activity by 40%, leading to calpain-mediated cleavage of ATP5A1 and a 50% decrease in ATP synthesis (23). Pharmacological inhibition of calpains with MDL28170 restored ATP5A1 expression and improved LVEF from 38% to 52% in septic mice (23).

Antioxidant therapies have shown promise in mitigating oxidative stress in SICM. Melatonin, a potent free radical scavenger, reduced LPS-induced myocardial MDA levels by 45% and increased SOD activity by 30% in rats, leading to a 2.2-fold increase in survival (24). Omaveloxolone, an Nrf2 activator, upregulated HO-1 and NQO1 expression by 2.5-fold and 3-fold, respectively, in LPS-treated H9C2 cells, reducing ROS levels by 50% and apoptotic cell death by 35% (25). Ferroptosis, an iron-dependent form of cell death, is also linked to oxidative stress in SICM: LPS-induced iron accumulation in H9C2 cells increased lipid peroxidation by 60%, while ferrostatin-1 treatment reduced myocardial neutrophil infiltration by 40% and improved LVEF by 20% in CLP-induced septic mice (26). These data underscore the critical role of oxidative stress in SICM pathogenesis and the therapeutic potential of antioxidant and ferroptosis-inhibiting agents.

### Genetic factors contributing to sepsis-induced cardiomyopathy

Genetic factors play a significant role in SICM susceptibility and severity, with both common variants and rare mutations influencing disease outcomes. Serum ferritin (SF) levels are a heritable risk factor: a Mendelian randomization study found that SF was associated with a 1.75-fold increased risk of cardiomyopathy (OR = 1.750, 95% CI: 1.152–2.657, p = 0.009) and a 3.08-fold increased risk of sepsis (OR = 3.079, 95% CI: 1.420–6.679, p = 0.004) (27). A case report shows that, genetic variants in sarcomere genes also contribute to SICM: pathogenic variants in TTN (titin) were identified in 25% of patients with dilated cardiomyopathy, a precursor to SICM (28). Additionally, PKCδ knockout mice were protected from CLP-induced cardiac dysfunction: LVEF remained at 62% vs. 48% in wild-type mice, and mitochondrial ROS levels were reduced by 50% (29). A prospective controlled study demonstrated that microRNAs (miRNAs) regulate gene expression in SICM: miR-150-5p in neutrophil-derived extracellular vesicles was upregulated by 2.1-fold in SICM patients, and multivariate logistic regression identified miR-150-5p as an independent predictor of SICM (AUC = 0.941) (30). These findings highlight the genetic basis of SICM and the potential for personalized risk stratification.

Epigenetic modifications and non-coding RNAs further modulate SICM susceptibility. Long non-coding RNA (lncRNA) MALAT1 alleviates LPS-induced myocardial injury by sponging miR-146a, leading to a 30% increase in SIRT1 expression and a 40% decrease in apoptotic cell death (31). Additionally, lncRNA MCM3AP-AS1 suppresses the miR-501-3p/CADM1/STAT3 axis, reducing inflammatory cytokine levels by 45% and improving LVEF by 25% in CLP-induced sepsis (32). These data suggest that epigenetic and non-coding RNA-mediated mechanisms are promising targets for SICM therapy.

## Epidemiology of sepsis-induced cardiomyopathy

### Incidence and prevalence of sepsis-induced cardiomyopathy

The incidence of SICM varies widely across studies due to heterogeneous diagnostic criteria, ranging from 13.8% in a Japanese tertiary care cohort to 50.3% in a Chinese multicenter study (2, 3). A meta-analysis of 16 studies reported a pooled prevalence of 20% (95% CI: 16–25%) in septic patients, with SICM associated with a 2.3-fold increased risk of short-term mortality (OR = 2.30, 95% CI: 1.43–3.69) (33). In a prospective cohort of 57 septic shock patients, 22.8% developed left ventricular dysfunction (LVEF < 50%), with complete recovery of LVEF (from 34.1% to 56.1%) in survivors (34). A retrospective analysis of the MIMIC-III database found an incidence of 28.2% (997/3530 patients) in sepsis patients without pre-existing cardiac disease, with SICM associated with higher hospital mortality (36.8% vs. 32.3% in non-SICM patients, p = 0.011) (35). These variations highlight the need for standardized diagnostic criteria to accurately estimate SICM burden. Temporal trends in SICM incidence reflect improvements in sepsis management and diagnostic techniques. A 20-year retrospective study in Taiwan found that the incidence of SICM increased from 18% in 2000 to 25% in 2011, likely due to enhanced detection with echocardiography (36). However, a prospective study in North India reported an incidence of 14% in septic ICU patients, with 50% of cases presenting with combined systolic and diastolic dysfunction (37). These epidemiological discrepancies reflect profound methodological heterogeneity beyond diagnostic thresholds. Key confounders include variable patient severity (ICU vs. general ward), geographic disparities in echocardiography access, temporal shifts in sepsis protocols (e.g., fluid resuscitation), and inconsistent exclusion of pre-existing cardiac disease. The Taiwanese temporal trend likely captures enhanced surveillance intensity rather than true incidence rise, while the Indian cohort’s lower rate may stem from resource constraints or distinct phenotypic expression. Notably, the meta-analysis (33) exhibits high statistical heterogeneity (I² > 70%), undermining the reliability of its pooled estimate. Crucially, without consensus on assessment timing (e.g., 24h vs. 72h post-admission), echocardiographic parameters, and adjustment for dynamic hemodynamic variables, reported SICM prevalence remains an artifact of study design—not biological reality. Standardization must integrate contextual factors to enable valid cross-study comparison and clinical translation, emphasizing the need for global epidemiological studies.

### Risk factors for sepsis-induced cardiomyopathy

Several clinical and laboratory factors are associated with an increased risk of SICM. Younger age (OR = 0.97 per year, 95% CI: 0.95–0.99), higher lactate levels (OR = 1.18 per mmol/L, 95% CI: 1.05–1.32), and pre-existing heart failure (OR = 3.77, 95% CI: 1.37–10.40) are independent risk factors in a Japanese cohort (2). In a MIMIC-III analysis, male sex (OR = 1.22, 95% CI: 1.05–1.42), diabetes (OR = 1.35, 95% CI: 1.12–1.63), and mechanical ventilation on ICU admission (OR = 1.51, 95% CI: 1.23–1.86) were associated with SICM (35). Additionally, low albumin levels (OR = 0.47 per g/dL, 95% CI: 0.23–0.93) and positive blood cultures (OR = 8.47, 95% CI: 2.24–55.61) predict SICM development in a retrospective study (38). These risk factors can be integrated into clinical scoring systems: a Chinese study developed a scoring system (range 0–6) based on age ≥87 years, NT-proBNP ≥3000 ng/L, respiratory rate ≥30/min, lactate ≥3 mmol/L, and SOFA ≥10 points, with a cut-off of 3 points yielding 71.4% sensitivity and 86.7% specificity for SICM diagnosis (39).

Sepsis severity and organ dysfunction also contribute to SICM risk. Patients with SOFA scores ≥10 have a 8.69-fold increased risk of SICM (95% CI: 2.541–29.742, p = 0.001) (39). In a prospective cohort of 145 septic patients, 50.3% developed SICM, with right ventricular dysfunction (12.4%) and left ventricular diastolic dysfunction (41.4%) being common phenotypes (3). These findings underscore the importance of early risk stratification to identify patients at high risk of SICM.

### Demographic variations in sepsis-induced cardiomyopathy

SICM exhibits significant demographic variations, with sex, age, and ethnicity influencing incidence and outcomes. Male septic patients have a 1.70-fold higher risk of ventricular arrhythmias (OR = 1.70, 95% CI: 1.50–1.94, p < 0.001) and a 28.2% higher incidence of SICM compared to females (2, 6). Age-related differences are also notable: younger patients (≤40 years) are more likely to develop left ventricular systolic dysfunction (LVSD) (42.86% vs. 16.4% in elderly patients), while elderly patients (≥65 years) have a higher prevalence of left ventricular diastolic dysfunction (57% vs. 23% in younger patients) (37, 40). Ethnic disparities exist in pediatric populations: African American and Hispanic children with cardiomyopathy have a 1.25-fold and 1.29-fold increased risk of in-hospital mortality, respectively, compared to white children (41). In adult patients, Asian sepsis survivors have a 19.5% mortality rate following VA-ECMO for SICM, compared to 61.0% in European patients (42). These demographic variations highlight the need for tailored diagnostic and therapeutic strategies.

Sex-specific differences in SICM pathophysiology further contribute to outcomes. Females with acute myocarditis (a precursor to SICM) are older (median 49.7 vs. 38.3 years, p < 0.001) and have higher NT-proBNP levels (1180 vs. 387 ng/L, p = 0.015) compared to males (43). Additionally, female gender is an independent predictor of in-hospital mortality in myocarditis patients (OR = 1.69, 95% CI: 1.1–2.6, p = 0.007) (44). These findings suggest that sex hormones and genetic factors may modulate SICM susceptibility and severity.

## Diagnostic approaches for sepsis-induced cardiomyopathy

### Biomarkers for early detection of sepsis-induced cardiomyopathy

Biomarkers play a crucial role in the early detection and risk stratification of SICM. Cardiac troponins and natriuretic peptides are the most widely used: a meta-analysis found that troponin I levels ≥0.21 μg/L and NT-proBNP levels ≥3000 ng/L are independent predictors of SICM (OR = 2.708, 95% CI: 1.093–6.711, p = 0.031) (39). Emerging biomarkers include microRNAs and long non-coding RNAs: miR-324-3p is upregulated by 2.3-fold in SICM patients, and its combination with echocardiographic parameters (LVEF, LVDd) improves diagnostic accuracy (AUC = 0.92 vs. 0.78 for echocardiography alone) (45). HIF-1α, a hypoxia-inducible transcription factor, is downregulated by 40% in SICM patients and is associated with a 3.5-fold increased risk of mortality (p = 0.009) (46). Additionally, cuproptosis-related biomarkers such as PDHB and DLAT are reduced by 35% and 28%, respectively, in SICM mouse models, with PDHB exhibiting high diagnostic accuracy (AUC = 0.995) (47). These biomarkers offer complementary information to traditional markers and may facilitate early intervention.

Inflammatory and oxidative stress biomarkers also aid in SICM diagnosis. Serum histone H4 levels ≥0.24 mg/L have a 65.2% sensitivity and 68.9% specificity for SICM patients (AUC = 0.684) (19). Ferroptosis markers such as GPX4 and SIRT1 are reduced by 45% and 30% in SICM patients, respectively, and their levels correlate with LVEF (r = 0.62, p < 0.001) (48). Collectively, SICM biomarkers span a utility continuum: established markers (troponin, NT-proBNP) offer accessible yet non-specific risk stratification, while emerging candidates (e.g., miR-324-3p, cuproptosis/ferroptosis-related proteins) provide mechanistic insights but lack analytical validation, assay standardization, multicenter replication, and proven impact on clinical decision-making. These findings highlight the potential of multi-biomarker panels to improve SICM detection.

### Imaging techniques in diagnosing sepsis-induced cardiomyopathy

Advanced imaging techniques are essential for the accurate diagnosis of SICM, particularly in patients with preserved LVEF. Speckle-tracking echocardiography (STE) detects subclinical myocardial dysfunction: global longitudinal strain (GLS) ≤-14.5% in septic shock patients has a 85% sensitivity and 78% specificity for SICM, compared to 62% sensitivity for LVEF <50% (8, 49). A meta-analysis of 5 studies found that GLS is significantly reduced in septic patients (-14.5% vs. -18.3% in controls, p < 0.001) and correlates with mortality (OR = 1.82 per 1% decrease in GLS, 95% CI: 1.23–2.69) (8). Cardiovascular magnetic resonance (CMR) imaging provides detailed tissue characterization: T2 mapping in septic patients with LVEF <50% shows increased myocardial edema (60.8 vs. 52.2 ms, p = 0.02), while extracellular volume (ECV) mapping reveals increased myocardial fibrosis (27.9% vs. 22.7%, p < 0.01) (50). Additionally, myocardial calcification, a rare complication of severe sepsis, can be detected by computed tomography (CT) in 3% of patients, with calcifications developing within 6 weeks of sepsis onset (51). These imaging modalities complement each other and improve diagnostic accuracy.

Point-of-care ultrasound (POCUS) is a valuable tool for bedside diagnosis of SICM. Hand-held ultrasound devices can detect LVSD (sensitivity 82%, specificity 76%) and right ventricular dysfunction (sensitivity 78%, specificity 81%) in septic patients (52). A multicenter prospective study found that POCUS phenotypes (e.g., hyperdynamic LV, hypodynamic LV) are associated with 28-day mortality (OR = 2.12 for hypodynamic LV, 95% CI: 1.34–3.35) (53). POCUS has transformed SICM detection at the bedside, with simplified protocols enabling rapid assessment of ventricular function even by non-cardiologists. However, a significant implementation gap exists between research recommendations and clinical practice. Most frontline clinicians rely on eyeball LVEF estimation despite its load-dependency. Bridging this gap requires development of simplified, validated POCUS algorithms incorporating strain imaging that can be deployed across diverse ICU settings.

### Clinical criteria for sepsis-induced cardiomyopathy diagnosis

The lack of standardized clinical criteria for SICM hinders its diagnosis and management. The most widely used definition includes a new-onset decrease in LVEF ≥10% to <50% with ventricular dilatation, reversible within 7–10 days (4). However, recent studies have expanded this definition to include diastolic dysfunction and right ventricular involvement: a Chinese study defined SICM as LVEF <50%, E/e’ ≥15, or right ventricular systolic dysfunction (tricuspid annular plane systolic excursion <15 mm) (3). A consensus statement proposed that SICM should be diagnosed in septic patients with: (1) new-onset myocardial dysfunction (systolic/diastolic/right ventricular), (2) no evidence of acute coronary syndrome, and (3) reversibility within 2 weeks. These criteria are supported by clinical data: a retrospective study of 359 septic patients found that SICM diagnosed by these criteria was associated with a 4.46-fold increased risk of in-hospital mortality (OR = 4.46, 95% CI: 1.15–18.69, p = 0.03) (38).

**Figure 1:**
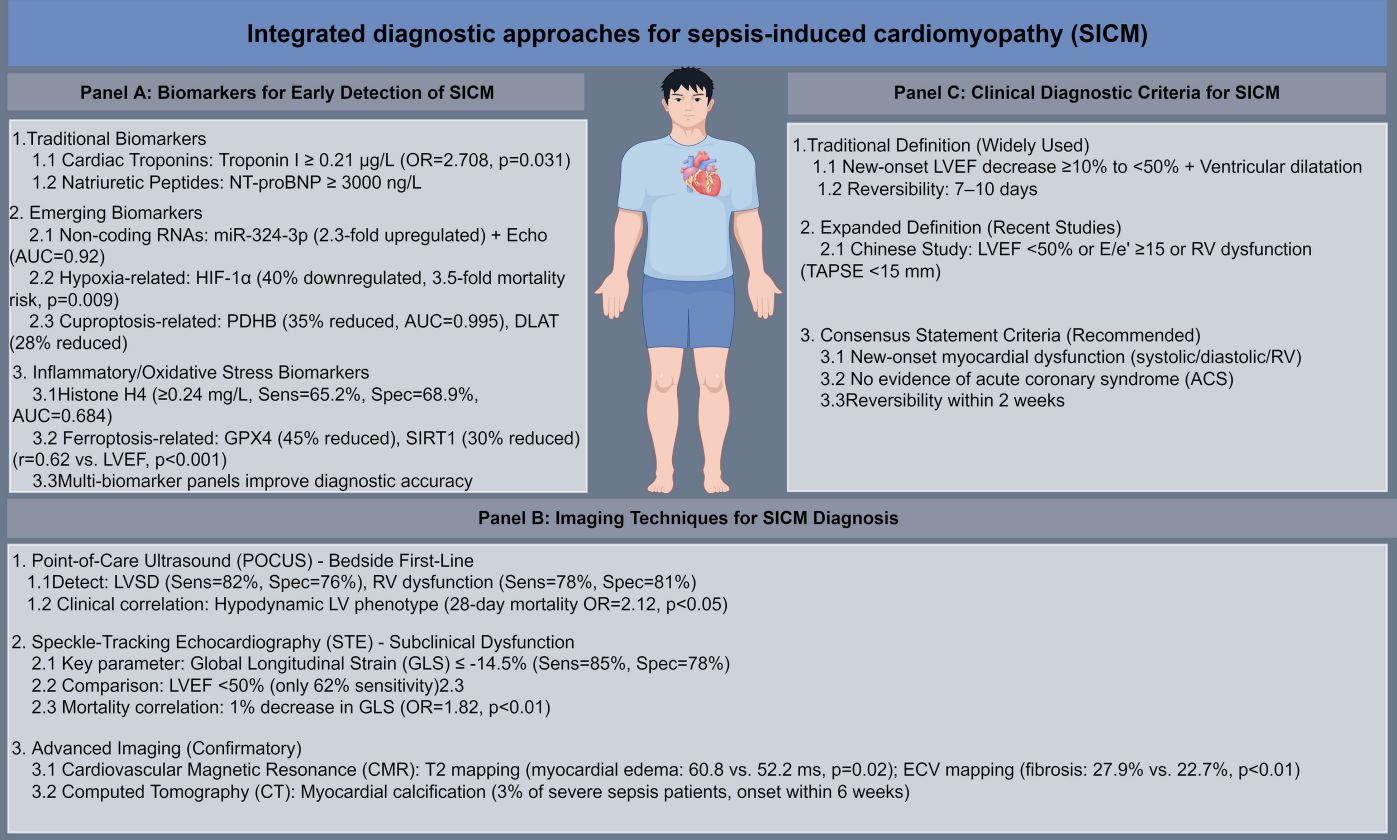
Integrated diagnostic approaches for sepsis-induced cardiomyopathy (SICM). **(A)** Key biomarkers for early detection of SICM, categorized into traditional markers (cardiac troponins, natriuretic peptides), emerging markers (non-coding RNAs, HIF-1α, cuproptosis-related proteins), and inflammatory/oxidative stress markers (histone H4, ferroptosis-related proteins). Critical cut-off values, fold changes, and diagnostic performance (AUC, OR, *p*-values) are indicated. **(B)** Hierarchical imaging techniques for SICM diagnosis, including bedside point-of-care ultrasound (POCUS) as first-line assessment, speckle-tracking echocardiography (STE) for subclinical myocardial dysfunction, and advanced cardiovascular magnetic resonance (CMR) and computed tomography (CT) for tissue characterization. Sensitivity, specificity, and clinical correlations are provided. **(C)** Evolution of clinical diagnostic criteria for SICM, from the traditional definition (LVEF decrease + ventricular dilatation) to expanded definitions (including diastolic and right ventricular dysfunction) and consensus criteria (three core requirements).

Challenges in SICM diagnosis include distinguishing it from other forms of cardiomyopathy (**Supplementary Table 1**). Takotsubo syndrome (TTS) is a common mimic: 46% of TTS patients have sepsis as a trigger, and they exhibit higher in-hospital mortality (40% vs. 25% in non-septic TTS patients) (54). Echocardiographic features such as apical ballooning (70% in TTS vs. 20% in SICM) and preserved right ventricular function (85% in TTS vs. 40% in SICM) help differentiate the two conditions (54, 55). These findings emphasize the need for careful clinical and imaging correlation in SICM diagnosis (**Figure 1**).

## Treatment strategies for sepsis-induced cardiomyopathy

### Pharmacological interventions for sepsis-induced cardiomyopathy

Pharmacological interventions for SICM focus on improving cardiac contractility, reducing inflammation, and mitigating oxidative stress. Levosimendan, a calcium sensitizer, improves cardiac index by 25% (from 2.2 to 2.8 L/min/m²) and reduces lactate levels by 30% in septic patients, may yield superior hemodynamic stabilization and lower acute kidney injury incidence versus dobutamine, though a meta-analysis found no significant mortality benefit (OR = 0.89, 95% CI: 0.69–1.16, p = 0.39), Current consensus emphasizes individualized therapy guided by echocardiography, lactate trends, and perfusion markers. Levosimendan may be preferentially considered in patients with microcirculatory dysfunction or high renal risk (8, 56). Melatonin, an antioxidant, reduces myocardial MDA levels by 45% and increases SOD activity by 30% in LPS-treated rats models, leading to a 2.2-fold increase in survival (24). Linagliptin, a DPP-4 inhibitor, inhibits NF-κB activation by 40% in HFD-CLP mice models, reducing myocardial iNOS expression by 35% and improving LVEF from 38% to 52% (57). Additionally, α_2_A-adrenergic blockade with BRL44408 increases cardiac norepinephrine levels by 50% in CLP rats models, leading to a 30% improvement in dP/dt and a 25% decrease in myocardial TNF-α levels (58). These agents target distinct pathophysiological pathways and may have synergistic effects.

Anti-inflammatory therapies also show promise in SICM. Sepsis-induced activation of the NLRP3 inflammasome is inhibited by MCC950, which reduces IL-1β levels by 60% and improves LVEF from 36% to 50% in CLP mice models (59). Apelin-13, a peptide hormone, attenuates TLR4 and NLRP3 signaling in septic rats, leading to a 40% reduction in myocardial inflammatory cell infiltration and a 2.5-fold increase in survival (60). These findings support the use of targeted anti-inflammatory agents in SICM.

### Role of mechanical support in sepsis-induced cardiomyopathy

Mechanical circulatory support (MCS) is reserved for severe SICM refractory to pharmacological therapy. VA-ECMO improves survival in SICM patients with LVEF <20% (62.0% vs. 32.1% in patients with LVEF >35%, p = 0.05) (42). A case report described successful use of an Impella device for 7 days in a patient with septic shock and SICM patients, with LVEF improving from 25% to 55% (61). Intra-aortic balloon pumping (IABP) is effective in inotrope-resistant SICM: a case series of 11 patients found that IABP increased cardiac output by 30% and reduced dobutamine requirements by 45% (62). However, MCS is associated with complications: sepsis is a major cause of death in patients with ventricular assist devices (21.2% in a pediatric cohort) (63). Careful patient selection is critical: VA-ECMO is most beneficial in patients with severe LVSD and no significant comorbidities (42).

### Emerging therapies for sepsis-induced cardiomyopathy

Emerging therapies target novel pathophysiological pathways in SICM, including ferroptosis, mitochondrial function, and epigenetic regulation. Ferrostatin-1, a ferroptosis inhibitor, reduces myocardial neutrophil infiltration by 40% and improves LVEF by 20% in CLP-induced sepsis (26). Mitochondrial-targeted therapies such as α-ketoglutarate normalize mitochondrial membrane potential and increase ATP synthesis by 35% in LPS-treated cardiomyocytes (64). Epigenetic modifiers such as engineered exosomes deliver miR-146a to septic hearts, reducing NF-κB activation by 40% and improving LVEF from 38% to 52% (64). Additionally, Chinese herbal medicines such as Xiaochaihu decoction (XCHD) inhibit ZBP1-initiated PANoptosis in septic mice, leading to a 30% decrease in myocardial apoptotic cell death (65). These therapies represent promising avenues for future research.

## Historical and current perspectives on sepsis-induced cardiomyopathy

### Evolution of understanding in sepsis-induced cardiomyopathy

The understanding of SICM has evolved significantly over the past five decades. Initially described as a rare complication of sepsis in 1984 (66), SICM is now recognized as a major contributor to sepsis mortality, with a prevalence of up to 50% (3). Early research focused on inflammatory cytokines as the primary drivers of myocardial dysfunction (3), but recent studies have highlighted the role of mitochondrial dysfunction, oxidative stress, and epigenetic modifications (5, 46). The introduction of advanced imaging techniques such as STE and CMR has improved the detection of subclinical SICM (49, 50), while multi-omics studies have identified novel biomarkers and therapeutic targets (46, 67). These advances have transformed SICM from a poorly understood condition to a well-characterized syndrome with targeted therapeutic strategies.

### Current clinical practices in managing sepsis-induced cardiomyopathy

Current clinical practices for SICM focus on hemodynamic stabilization, infection control, and supportive care. The Surviving Sepsis Campaign recommends fluid resuscitation to achieve a CVP of 8–12 mmHg, followed by vasopressors to maintain MAP ≥65 mmHg (68). Inotropes such as dobutamine are used for patients with cardiac dysfunction and persistent hypoperfusion, though levosimendan may be preferred in patients with β-adrenergic receptor desensitization (9). Early use of invasive hemodynamic monitoring with a pulmonary artery catheter (PAC) is associated with lower mortality in sepsis-induced cardiogenic shock (OR = 0.58, 95% CI: 0.46–0.72, p < 0.001) (69). Additionally, a multidisciplinary approach involving a cardiogenic shock team improves survival in severe SICM (55% vs. 30% in standard care, p = 0.02) (68). These practices are supported by clinical evidence and form the basis of current guidelines.

### Recent advances in sepsis-induced cardiomyopathy research

Recent advances in SICM research include the identification of novel pathophysiological mechanisms and therapeutic targets. Single-cell RNA sequencing has revealed that macrophages are the predominant immune cell type in septic hearts, with ITGAM modulating their recruitment and polarization (70). Multi-omics studies have identified efferocytosis-related genes (CEBPB, CD36) as diagnostic biomarkers, with tamibarotene showing potential as a therapeutic agent (71). Additionally, the role of PANoptosis in SICM has been elucidated: XCHD inhibits ZBP1-initiated PANoptosis, leading to a 30% decrease in myocardial apoptotic cell death (65). These advances have expanded our understanding of SICM and opened new avenues for research.

## Future directions and controversies in sepsis-induced cardiomyopathy

### Potential therapeutic targets in sepsis-induced cardiomyopathy

Future therapeutic targets in SICM include mitochondrial function, ferroptosis, and epigenetic regulation. Mitochondrial dynamics modifiers such as P110, which inhibits Drp1/Fis1 interaction, normalize mitochondrial membrane potential and improve cardiac function in septic mice (22). Ferroptosis inhibitors such as ferrostatin-1 reduce lipid peroxidation and improve survival in CLP-induced sepsis (26). Epigenetic modifiers such as lncRNA MCM3AP-AS1 suppress the miR-501-3p/CADM1/STAT3 axis, reducing inflammatory cytokine levels by 45% (32). Additionally, targeting the Mst1/Drp1/mitochondrial fission/F-actin axis with Mst1 inhibitors reduces cardiomyocyte death by 35% in LPS-treated hearts (72). Despite promising preclinical data on targeted interventions (e.g., ferroptosis inhibitors, mitochondrial stabilizers), critical translational gaps persist. No phase II/III randomized trials have evaluated ferroptosis inhibitors in human SICM, and mitochondrial-targeted therapies remain confined to animal models with questionable clinical relevance to heterogeneous septic populations.

### Controversial issues in sepsis-induced cardiomyopathy management

Several controversies persist in SICM management, including the role of inotropes, MCS selection, and diagnostic criteria. The use of dobutamine vs. levosimendan in SICM remains debated: a meta-analysis found no mortality benefit for levosimendan (OR = 0.89, 95% CI: 0.69–1.16, p = 0.39), though it improves cardiac index (56). The choice of MCS (Impella vs. VA-ECMO) depends on patient characteristics: Impella is preferred for isolated left ventricular dysfunction, while VA-ECMO is used for biventricular failure (42). Standardized diagnostic criteria for SICM are lacking, with variations in LVEF thresholds and inclusion of diastolic dysfunction. The mismatch between preclinical homogeneous animal models and clinical heterogeneous patient populations; the inconsistent efficacy of anti-inflammatory agents in animal experiments versus clinical trials; and the lack of standardized translational pathways for ferroptosis inhibitors and mitochondrial-targeted therapies. These controversies highlight the need for large-scale randomized controlled trials to establish evidence-based practices.

### Future research directions in sepsis-induced cardiomyopathy

Future research directions in SICM include personalized medicine, multi-omics integration, and targeted therapeutics. Genomic studies to identify SICM susceptibility loci may enable risk stratification and personalized treatment (27). Multi-omics approaches combining transcriptomics, proteomics, and metabolomics will identify novel biomarkers and therapeutic targets (67). Additionally, clinical trials of emerging therapies such as ferroptosis inhibitors and mitochondrial modifiers are needed to validate their efficacy (22, 26). Finally, the development of standardized diagnostic criteria and treatment guidelines will improve consistency in SICM management. These research priorities will advance our understanding of SICM and improve patient outcomes.

### Quality assessment of included studies

To ensure methodological rigor, clinical observational studies (e.g., MIMIC-III database analyses, multicenter Chinese cohorts) were assessed using the Newcastle-Ottawa Scale (NOS), while animal experiments underwent bias risk evaluation via SYRCLE’s risk of bias tool. Assessments were performed independently by two reviewers, with discrepancies resolved by a third arbitrator. Quality ratings for pivotal studies are summarized in **Supplementary Table 2**.
