# Supplementary material for: Sepsis-induced cardiomyopathy: mechanisms, epidemiology, diagnosis, and treatments
Source: Front Immunol. 2026 Mar 11;17:1785463. doi: 10.3389/fimmu.2026.1785463 (PMC13012987; doi:10.3389/fimmu.2026.1785463)
Supplement: Supplementary file 1 [file Table1.pdf]

Table 1. Differential Diagnosis - SICM vs. Takotsubo Syndrome (TTS)

Features	Takotsubo Syndrome (TTS)	Sepsis-Induced Cardiomyopathy (SICM)
Sepsis as trigger	46% of cases	100% of cases
Echocardiography	Apical ballooning (70%)	Apical ballooning (20%)
Right ventricular function	Preserved (85%)	Impaired (60%)
In-hospital mortality	40% (septic TTS)	25% (non-septic TTS reference)
