# Supplementary material for: Sepsis-induced cardiomyopathy: mechanisms, epidemiology, diagnosis, and treatments
Source: Front Immunol. 2026 Mar 11;17:1785463. doi: 10.3389/fimmu.2026.1785463 (PMC13012987; doi:10.3389/fimmu.2026.1785463)
Supplement: Supplementary file 2 [file Table2.pdf]

Table 2. Quality and Bias Risk Ratings of Key Included Studies

Study [Author, Year]	Design	Tool	Rating	Key Strengths	Key Limitations
Sato et al. (2016)	Retrospective cohort	NOS (9/9)	High quality	Representative cohort, stringent exclusions, complete follow-up	Incomplete confounder adjustment, echocardiography coverage 67.9%
Guan et al. (2022)	Multicenter prospective	NOS (6/9)	Moderate quality	Systematic early echocardiography, diastolic parameter inclusion	Potential selection bias, less rigorous pre-existing cardiac condition exclusion
Liang et al. (2021)	MIMIC-III analysis	NOS (8/9)	High quality	Large sample (n=3,530), multivariable adjustment	Retrospective constraints, limited echo subgroup data
Bansal et al. (2023)	Prospective observational	NOS (7/9)	Moderate quality	Standardized point-of-care ultrasound protocol	Single-center, modest sample (n=120)
Wang et al. (2017) to Chen et al. (2018)	Rodent models (LPS/CLP)	SYRCLE	Low/Moderate risk	Random allocation, blinded assessment, predefined endpoints (where applicable)	Inadequate allocation concealment reporting, limited clinical translatability, variable housing standardization

NOS interpretation: ≥ 8 = high quality; 6–7 = moderate; ≤ 5 = low. SYRCLE: $\geq 70\%$ low-risk domains = low bias risk. Heterogeneity in SICM diagnostic criteria and population severity contributes to variable clinical evidence quality. Common limitations across preclinical studies include insufficient reporting of randomization concealment and blinding procedures.
